# Using Stock-Flow Diagrams to Visualize Theranostic Approaches to Solid Tumors in Personalized Nanomedicine

**DOI:** 10.3389/fbioe.2021.709727

**Published:** 2021-07-22

**Authors:** Virginia Cazzagon, Alessandra Romano, Francesco Gonella

**Affiliations:** ^1^Department of Environmental Sciences, Informatics and Statistics, University Ca’ Foscari of Venice, Venice, Italy; ^2^Department of Molecular Sciences and Nanosystems, University Ca’ Foscari of Venice, Venice, Italy; ^3^Scuola Superiore di Catania, Università degli Studi di Catania, Catania, Italy; ^4^Department of Molecular Sciences and Nanosystems, Ca’ Foscari University of Venice, Venice, Italy; ^5^Research Institute for Complexity, University Ca’ Foscari of Venice, Venice, Italy

**Keywords:** iron oxide nano-biomaterials, system thinking, theranostic, personalized nanomedicine, complex systems, nanotechnology

## Abstract

Personalized nanomedicine has rapidly evolved over the past decade to tailor the diagnosis and treatment of several diseases to the individual characteristics of each patient. In oncology, iron oxide nano-biomaterials (NBMs) have become a promising biomedical product in targeted drug delivery as well as in magnetic resonance imaging (MRI) as a contrast agent and magnetic hyperthermia. The combination of diagnosis and therapy in a single nano-enabled product (so-called theranostic agent) in the personalized nanomedicine has been investigated so far mostly in terms of local events, causes-effects, and mutual relationships. However, this approach could fail in capturing the overall complexity of a system, whereas systemic approaches can be used to study the organization of phenomena in terms of dynamic configurations, independent of the nature, type, or spatial and temporal scale of the elements of the system. In medicine, complex descriptions of diseases and their evolution are daily assessed in clinical settings, which can be thus considered as complex systems exhibiting self-organizing and non-linear features, to be investigated through the identification of dynamic feedback-driven behaviors. In this study, a Systems Thinking (ST) approach is proposed to represent the complexity of the theranostic modalities in the context of the personalized nanomedicine through the setting up of a stock-flow diagram. Specifically, the interconnections between the administration of magnetite NBMs for diagnosis and therapy of tumors are fully identified, emphasizing the role of the feedback loops. The presented approach has revealed its suitability for further application in the medical field. In particular, the obtained stock-flow diagram can be adapted for improving the future knowledge of complex systems in personalized nanomedicine as well as in other nanosafety areas.

## Introduction

In the last years, the use of nano-biomaterials (NBMs) has led to great improvements in several biomedical applications such as diagnostic, therapeutic, and regenerative medicine (Wang et al., 2018). In particular, iron oxide NBMs have been used in a large variety of biomedical applications such as diagnostics, imaging, hyperthermia, magnetic separation, cell proliferation, photodynamic therapy, tissue repair, and drug delivery (Bruschi and de Toledo, 2019; Dadfar et al., 2019), thanks to their suitable structural, colloidal, and magnetic properties as well as their negligible toxic effects (Ghazanfari et al., 2016; Aires et al., 2017).

In the oncologic context, magnetite (Fe_3_O_4_) NBMs can be used as contrast agent in magnetic resonance imaging (MRI) for diagnosis purposes, while in therapeutic nanomedicine they can be accumulated in cancer cells through the enhanced permeability and retention effect (Nuzhina et al., 2019), then generating heat upon the application of an alternate magnetic field (MF) in hyperthermia treatments (Vallabani and Singh, 2018). The combination of therapeutic and diagnostic capabilities using a single nano-based biomedical product, the so-called nanotheranostic (Theek et al., 2014), addresses the administration of Fe_3_O_4_ NBMs to (i) obtain *in vivo* imaging of the tumor site, (ii) treat the tumor site after the target drug delivery, and (iii) induce cancer cell death by hyperthermia.

However, as some nanotheranostic agents are currently at Phase I and Phase II clinical trials (Singh et al., 2020; Verry et al., 2020), there is the urgent need to investigate not only the safety profile of nanotheranostics in both early and advanced phases of clinical trials (Singh et al., 2020) but also to understand how these innovative products can be personalized, considering inter-individual variability in therapy selection, treatment planning, objective response monitoring, and follow-up therapy planning based on the specific characteristics of the tumor tissue (Ryu et al., 2014; Keek et al., 2018; Degrauwe et al., 2019). Indeed, as exhaustively explained in Bielekova et al. (2014), systems biology principles represent a unique opportunity to predict complex diseases in comparatively small cohorts of patients through the identification of functional networks at the organism/patient-level. Moreover, as health systems are self-organizing and tightly linked, constantly changing and governed by positive or negative feedbacks (WHO, 2009), there is the need to identify and represent their complexity in a holistic perspective.

Systems Thinking (ST) approach shifts the attention from the study of local events, in terms of causes, effects, and mutual relationships, to the study of the systemic patterns from which they emerge, describing the change in the hierarchical feedbacks structure that gives access to the operational configurations of the system as a whole. In the ST context, analytical tools based on stocks and flows representations have been developing since the 70s by Jay Forrester at the Massachusetts Institute of Technology mainly focused on Social Systems (Forrester, 1971). Afterward, ST approaches have found application in several other fields, as reported, for example for business (Sterman, 2000), energy and sustainability (Higgins, 2015; Kutty et al., 2020), ecology (Assaraf and Orion, 2005), biogeochemistry (Haraldsson and Sverdrup, 2013), communication (Gonella et al., 2020), and medicine (Romano et al., 2021). Nevertheless, the use of ST approach in nanomedicine-related studies is still lacking.

As a matter of fact, nanotheranostic systems have started demonstrating their efficacy in diagnosis but lack therapeutic competence and vice versa (Alshehri et al., 2021). In particular, even when diagnostic and therapeutic protocols are separately well-established, mutual interference between them may arise when used together. For example, diagnostic and therapeutic procedures that operate at even slightly different temporal and spatial scales may compromise the effectiveness of the theranostic procedure. For this reason, the adoption of a ST approach can help in the identification and the choice of the best temporal and spatial scales for the whole theranostic protocol. Furthermore, the systemic overall reaction to the change of external parameters or driving forces (e.g., administration of other drugs) might be addressed through the analytical stock-flow representation.

In this work, a ST stock-flow diagram is developed for the first time to represent the complexity of the use of Fe_3_O_4_ NBMs as a theranostic agent in solid tumors based on the personalized nanomedicine perspective. In particular, the magnetite case study consists of (1) a magnetic core of Fe_3_O_4_ NBMs coated with polyethylene glycol (PEG) and poly co-glycolic acid (PLGA), (2) a sustained released anticancer drug, and (3) immune system (IS) cell loading. This product can be classified as Advanced Therapy Medical Products (ATMPs), which constitute a class of innovative pharmaceuticals based on emerging cellular and molecular biotechnologies for somatic cell therapy [Regulation (EC) No 1394/2007] and patient-specific products.

## Materials and Methods

### System Thinking Approach and Its Elements

As extensively described in Odum and Odum (2000), the comprehensive ST approach includes the development of three scales of modeling:

1. Structural graphic model, in which the fundamental structure that determines the system dynamics is diagrammed in terms of stocks, flows, and processes.
2. Analytical model, in which formal relationships are established between the system’s components, allowing to define a set of differential equations able to describe the systemic behavior even for situations difficult to observe experimentally.
3. Computational model, which transforms the set of interconnected differential equations into a simulator, studying how the system dynamics is affected by a change in external parameters, driving forces, perturbations, or, in the case of disease systems, the application of specific therapies.

In this work, we present for the first time a structural graphic model for the representation of theranostic modalities of a nano-based biomedical product. The development of this model is the first step for the other two, which will be made possible when clinical data on the administration of magnetite NBMs containing anticancer drug used as theranostic agent start to become available. In the following sections, an introduction of the structural ST diagram and its application to the investigated case study is presented, to guide the reader through the final diagram.

The structural stock-flow representation of the ST-based approach is set up following the procedure:

1. Identification of a set of stocks.
2. Choice of a proper boundary.
3. Identification of the flows connecting the stocks, also with the external environment.
4. Identification of the processes occurring within a system.

**FIGURE 1:**
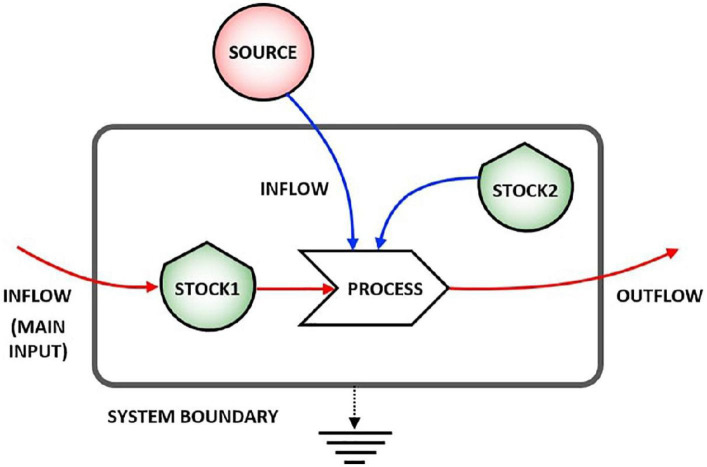
Representation of a stock-flow system. Stock 1 undergoes a process due to the action of both Stock 2 and an external source.

Figure 1 shows the main symbols used in stock-flow diagrams based on the energy language (Odum and Odum, 2000), where shields indicate the stocks, line arrows the flows, and solid arrows the processes that are always activated or controlled by a stock inside or outside the system (i.e., arrow coming from outside the system), and the smooth gray rectangle the system boundary. For the second principle of thermodynamics, energy is partially lost in any physical process, and this is represented by the flow going down to the earth symbol (heat sink).

#### The Stocks

Stocks are elements represented by an extensive variable (i.e., material, energy, and information). A stock changes over time only through the action of flows (i.e., inflows and/or outflows), and may therefore act as delay or buffer or shock adsorber for the system (Sterman, 2000). The stock content must be countable extensive state variables *Q_*i*_ i* = 1, 2, …, *n*, that constitute an *n*-tuple of numbers that at any time represents the state of the system. The choice of the set of variables depends on the hierarchical level of the desired description, as well as on the overall purpose of the study.

Stocks must be chosen respecting some requirements:

1. The number of the stocks must be as low as possible to describe the state of the system for the prescribed purposes.
2. It must be possible to describe any relevant macroscopic in terms of stocks interactions.
3. Any system change (either detectable from the external or not) must correspond to a change in the n-tuple of state variables.
4. Stocks should be measurables, or at least a set of plausible values at a certain time should be conceivable, to study their evolution.

A stock may represent either a physically located set of a variable, or a virtual set of elements that play a specific role in the system dynamics, even without having the corresponding location in the real space.

The choice of the stocks relevant for the case at issue is a fundamental step in the stock-flows approach. When clinical data are available, a value at *t*_0_ must be assigned to each stock in order to make a quantitative analysis. The determination of these initial values can be performed by either directly measuring them or by determining a “plausibility interval.” In this latter case, a sensitivity analysis is performed to validate the model testing the system response within the selected interval of values.

#### The Boundary

A proper choice and definition of the systemic boundary is an important task since the boundary defines the objective of the systemic study depending on the main inflows and the system outputs. In ST, the boundary is an abstract element, possibly extended in both space and time, and has the main role of isolating the elements which are necessary to give an exhaustive description of the dynamics of the system at the chosen level of the study and to focus on the relationships between the internal elements (Brown, 2004). The choice of the boundary will reflect also the hierarchical level of the feedbacks that will depend on the time-span of the diagram description.

#### The Flows

In a stationary state of the system, stocks values are constant and may change their values through inflows and/or outflows, represented by arrows entering or exiting stocks and expressed as *dQ/dt*.

**FIGURE 2:**
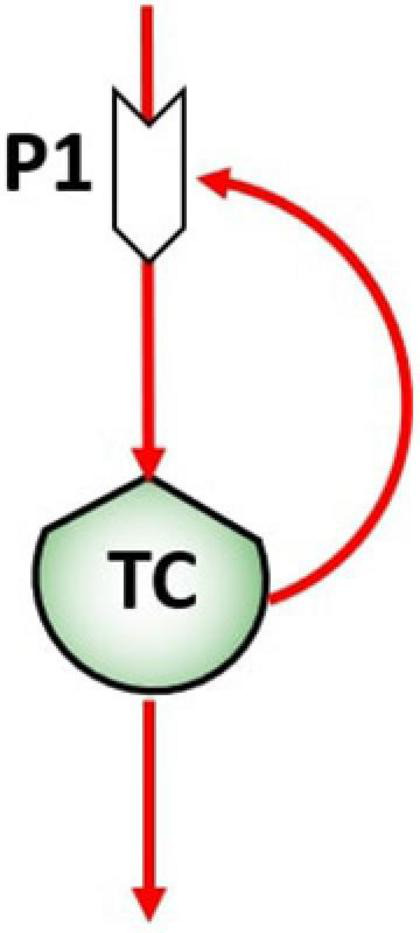
Reinforcing feedback of the tumor cells stock (TC) on the birth process (P1), giving rise to the proliferation.

In biological systems, flows can be flows of matter (energy), that constitute the mechanism by which a stock value may change in time, and flows of information, responsible for the control action exerted by stocks on the processes that in turn control the flows. The control flows network is a fundamental aspect in ST diagramming since their action is responsible for feedbacks and causal loop formation at different time scales (Haraldsson, 2004). The pattern of feedbacks is therefore the feature that defines the system dynamics. For example, the unregulated proliferation of tumor cells (TC) in the human body can be represented by reinforced feedback, as represented in Figure 2, when TC growing and multiplying in an uncontrolled manner (Cooper, 2000). An increase of TC in the stock will then determine an exponential proliferation of TC at time *t*_1_.

Flows are described by phenomenological coefficients that represent how much of the contribution from one or more stocks will be effective in their interaction on the process. Therefore, these coefficients represent the dynamics of the system to point out the interconnection network between its operational elements. In fact, it is important to underline that ST approach is not interested in representing the physical mechanisms of feedback controls, but in drawing the interactions among them. A detailed description of the conceptual basis of the quantitative setting up of stock-flow diagrams may be found for example in Odum and Odum (2000), where the counter-intuitive aspects of the approach are examined in many different systems.

#### The Processes

Processes represent the interaction between the stocks and determine the dynamics of the system. Processes are capable to alter – either quantitatively or qualitatively – a flow, by the action of one or more system elements. Since the system state is a collection of stock values, and the only way to change the value of a stock is by acting on its in/outflows, processes are located along the flow lines. In general, the location of a process in the diagram does not have any correspondence in a physical location in real space. Moreover, a process must be activated by another driver as flows of information or matter control the occurring processes and thus the value and/or nature of the flows.

### System Thinking Diagram of Theranostic Approach Combined With Personalized Nanomedicine of Solid Tumors Using Magnetite NBMs

Stocks, flows, and processes were selected based on information collected from the literature on the personalized nanomedicine and theranostics modalities of iron oxide NBMs. The descriptions of stocks and processes selected for the diagram on theranostic approach, combined with personalized nanomedicine of solid tumors using magnetite NBMs, are reported in Table 1. All the stocks are countable variables. Immune system (IS) and the bloodstream (BS) are regarded as systems since their action involves different variables which are not essential for the overall description of the system at issue. The MF and MRI are regarded as sources of energy and represented by a circle.

**TABLE 1:** Description of the selected stocks, processes, sources, and systems and their abbreviations used in the final diagram.

**Abbreviation**	**Type of element**	**Description**	**References**
IC	Stock	Immune cells intravenously collected from the patient	Galli et al., 2021
NBMs	Stock	Magnetite NBMs coated with PEG and PLGA	Ghazanfari et al., 2016
TC	Stock	Tumor cells	Galli et al., 2021
D	Stock	Anticancer drug	Douziech-Eyrolles et al., 2007
ATMP	Stock	ATMP is formed when IC, NBMs, and D are uptake by immune system	European Commission, 2007
ATMP + TC	Stock	ATMP bonded with TC	Mura and Couvreur, 2012
i	Stock	Medical knowledge related to (i) theranostic modalities and personalized nanomedicine, (ii) information collected from diagnosis, and (iii) information needed for the selection of specific therapy	Bielekova et al., 2014; Comte et al., 2020
ROS	Stock	Reactive oxygen species generated by the hyperthermia process and outside the system	Slimen et al., 2014; Aggarwal et al., 2019
IS	System	Immune system	Le et al., 2019
BS	System	Bloodstream	Le et al., 2019
MF	Source	Magnetic field	Mura and Couvreur, 2012
MRI	Source	Magnetic resonance imaging	Mura and Couvreur, 2012; Revia and Zhang, 2016
Injec.	Process	Injection administration	Mura and Couvreur, 2012
Upt.	Process	Uptake of NBMs from immune system	Mura and Couvreur, 2012
Activ.	Process	Activation/targeting of the ATMP on the TC	Dadfar et al., 2019
Hyp.	Process	Hyperthermia	Ansari et al., 2018; Dadfar et al., 2019
Bioim.	Process	Bioimaging of the TC	Mura and Couvreur, 2012; Revia and Zhang, 2016
Apop.	Process	Apoptosis of the TC	Goya et al., 2008; Hou et al., 2014; Jagtap et al., 2020

## Results

### System Thinking Diagram of Theranostic Approach Combined With Personalized Nanomedicine of Solid Tumors Using Magnetite NBMs

**FIGURE 3:**
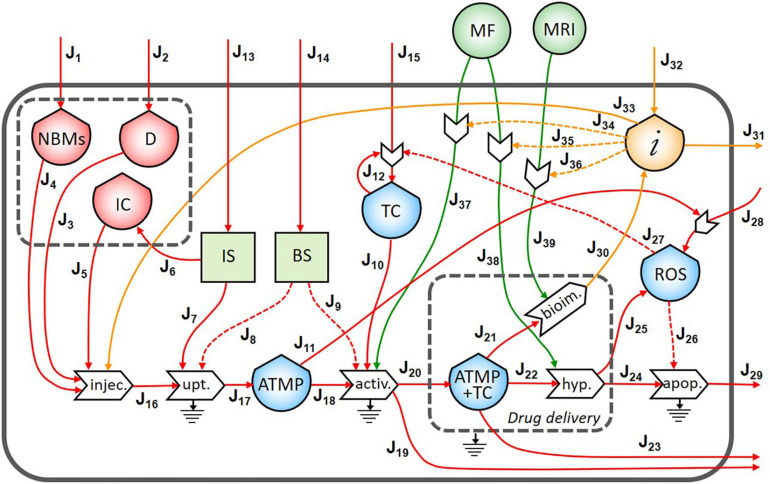
Representation of the theranostic approach of the magnetite nano-biomaterials (NBMs) to solid tumor.

In Figure 3, the final diagram representing the theranostic approach combined with personalized nanomedicine of solid tumors using magnetite NBMs coated with PEG and PLGA is presented. In red flows of mass, in green flows of energy, and yellow flows of information are represented, where dashed lines indicate the controls exerted by the stocks on the processes.

Nano-biomaterials (J1) and anticancer drug (J2) are the main inflows of the diagram. A specific quantity of NBMs (J4) and drug (J3) are intravenously administered together with immune cells previously sampled from the patient’s blood (J5). In healthy people, the IS plays important roles in controlling the growth of malignant cells while in cancer patients can even facilitate the growth of TC (Le et al., 2019). For this reason, a quantity of immune cells needs to be carefully sampled (J6) to provide an efficient uptake process. The uptake process transforms the injected medicinal product (J16) into one outflow represented by the ATMP bioavailable in the blood (J17), which is controlled and activated by both the IS (J7) and the BS (J8). Inflows of the BS and IS (J13 and J14) are coming from outside of the system. However, if the ATMP has low efficacy, the presence of the ATMP in the blood can activate the proliferation of reactive oxygen species (ROS) (J11) which may cause the activation of the proliferation of TC (Aggarwal et al., 2019). The stock of TC is formed by an inflow (J15) and a feedback loop (J12), which represents the uncontrolled proliferation of TC (as also represented in Figure 2). The accumulation of the ATMP (J18) at the tumor site (J10) is based on drawing it to the tumor site by using an external MF (Revia and Zhang, 2016) (J37) in the BS, that activates the targeting/activation process (J9). Depending on the efficacy of this ATMP, a small quantity of this product may undergo the clearance process by the reticuloendothelial system without reaching the tumor site (J19) (Yu and Zheng, 2015) and not all the ATMP at the tumor site may go through the hyperthermia or bioimaging processes (J23).

During the formation of the stock of ATMP and TC (J20), the release of anticancer drug at the tumor site is represented by the small gray box (drug delivery). Then, under alternating MF (J38), magnetite NBMs on the tumor site (J22) can transform the electromagnetic energy into heat (hyperthermia process) causing localized heating of the TC (J24) and thus triggering the commitment to apoptosis of cancer cells (Goya et al., 2008; Jagtap et al., 2020) and their death (J29). The apoptosis process of TC can be generated not only as a consequence of heating of tumor site but also activated by ROS production (Hou et al., 2014) (J25). Indeed, in the diagram, the commitment to apoptosis of TC is activated by the flow of ROS (J26). However, as some ROS can diffuse freely across cell membranes, they can mediate toxic effects far from the site of ROS production (J28) (Slimen et al., 2014), also activating the proliferation of TC (J27) (Aggarwal et al., 2019).

The formation of the stock of ATMP and TC (J21) can also permit to perform imaging of the tumor site and real-time treatment monitoring of therapeutic drug delivery using MRI (J39), thereby adjusting treatment methods (Revia and Zhang, 2016). Indeed, a flow of information is generated from the bioimaging process (J30) which constitutes, together with the medical knowledge of healthcare personnel (J32), the main inflow of the stock of information. All the collected information is then used to activate the hyperthermia process by setting the alternating MF properly (J35), depending on the morphological properties of the tumor tissues, (ii) to activate the following bioimaging process (J36), and (iii) to set the MF during the activation process of ATMP on TC (J34). Moreover, all the knowledge and considerations related to the inter and intra-patient variability are then used to define the quantity of the drug to be injected to increase the efficacy of the following therapy (Comte et al., 2020) (J33). Information collected during theranostic activities will be also used outside the system for further research (J31).

### Feedback Loops

In the ST diagram, five reinforced feedbacks were identified. The first is represented by the proliferation of the TC as explained in section “The Flows,” while the other four are related to the personalized nanomedicine concept.

**FIGURE 4:**
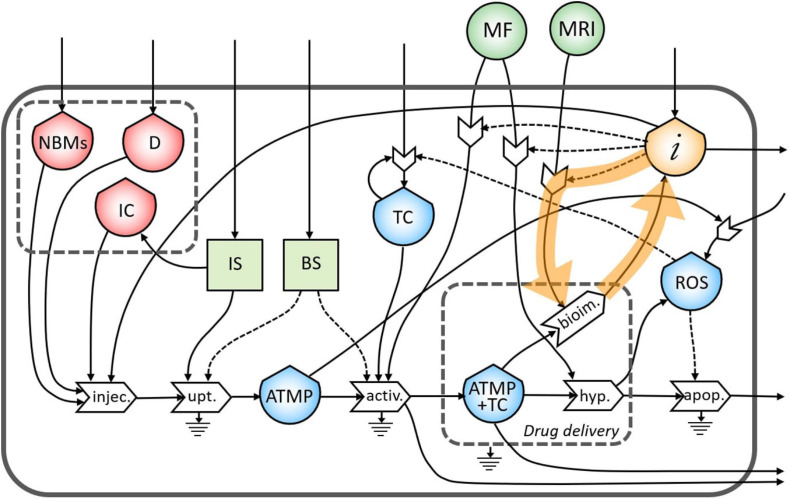
Representation of the feedback loop of the flow of information on the bioimaging process.

As underlined in Figure 4, the bioimaging process permits the generation of a flow of information related to the morphological characteristics of the tumor site. This flow creates a feedback loop of information necessary to tune the MRI operation itself.

**FIGURE 5:**
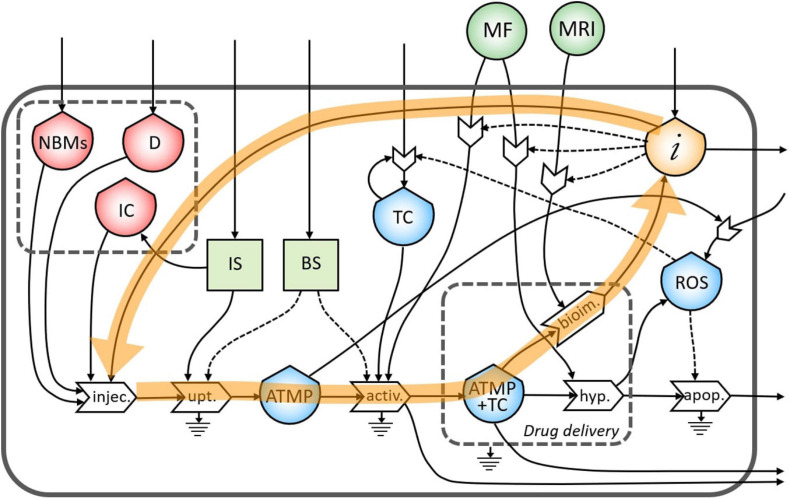
Representation of the feedback loop of the flow of information from the bioimaging to the injection process.

The same flow of information coming from bioimaging process is useful also to tune a subsequent administration of the ATMP depending on the morphological characteristics of the tumor site (Figure 5).

**FIGURE 6:**
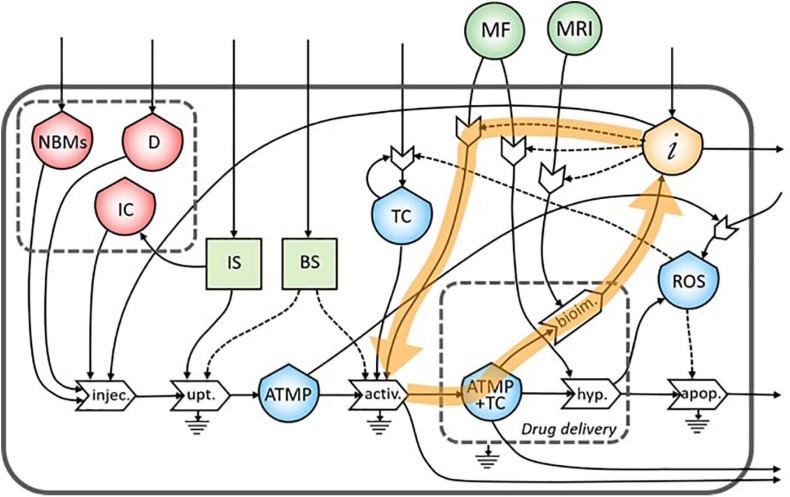
Representation of the feedback loop of the flow of information on the activation process.

Moreover, information collected during the bioimaging is extremely useful also during the targeting of the magnetic NBMs to the tumor site through the application of a specific MF, as underlined in Figure 6.

**FIGURE 7:**
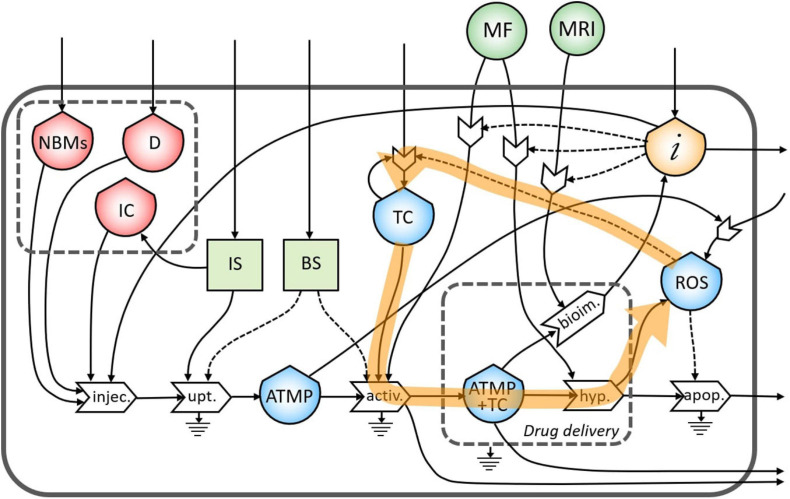
Representation of the feedback loop of the flow of reactive oxygen species (ROS) in the proliferation of TC.

During the treatment of the TC in the hyperthermia process, high levels of ROS are produced by the increased metabolic activity and mitochondrial dysfunction (Liou and Storz, 2010), which can lead to the proliferation of TC (Figure 7). This feedback loop represents the theranostic activities of this ATMP. Indeed, the correct administration of this product permits the identification of the tumor site as well as treating it minimizing the proliferation of TC.

## Discussion

This study represents the first application of the ST theory in personalized nanomedicine. More specifically, the ST approach has been considered to study the interconnection between diagnosis and therapy of solid tumors using a single nano-enabled biomedical product (so called nanotheranostics) (Theek et al., 2014) through the development of a stock-flow diagram.

The investigated nano-product is a not yet commercialized dispersion of Fe_3_O_4_ NBMs coated with PEG and PLGA containing an anticancer drug which may be classified as ATMP. In the near future, dynamics related to the application of such innovative medicinal products will need to be carefully investigated in order to define the most suitable and effective procedure for the selection of a patient-specific therapy.

During the past, several methods have been developed to quantify biological networks, for example, flux balance analysis (FBA) (Lee et al., 2006), metabolic flux analysis (Lagziel et al., 2019), and quantitative systems pharmacology (QSP) (Wang et al., 2020; Balti et al., 2021; Chelliah et al., 2021). However, the resulting dynamic systems are not sufficiently comprehensive for generating a large-scale model.

For this reason, in the current study, a ST top-down approach has been followed to represent the self-organized system, through which the global dynamics of the systemic patterns may be obtained using an analytical representation of the stocks, flows, and processes in different systemic time-scales. For the development of the presented diagram, no specific tools are used for the identification of flows and stocks. Indeed, the ST approach permits to develop several systems representing the same complex system but a different level of hierarchy.

The structure of the presented ST diagram forces the system toward a limited set of possible configurations at the selected level of complexity, from which important feedback loops emerge, as (i) how the personalized nanomedicine can help in the diagnosis and treatment of tumor sites, (ii) what could affect the proliferation of TC, and (iii) how the obtained information can help in the choice of subsequent treatments and/or diagnosis.

The strength of the presented ST diagram is its ability to clearly communicate the network of feedbacks. Indeed, the interconnections between stocks and flows may therefore shed some new light on how to manage the complexity of a disease, since a correct identification of the accessible dynamical patterns may allow finding the proper leverage points for intervening (Meadows, 2008), especially in the oncologic context. The whole complexity of anticancer nanomedicine was also suggested by Sun et al. (2020) where authors underline the need to carefully evaluate the efficacy of nano-enabled anticancer drugs considering the tumor heterogeneity from a systemic point of view as otherwise, benefits could not outweigh adverse effects. Moreover, the ST diagram demonstrates the differences between personalized medicine and traditional medicine by the stock and flow of information. Indeed, the reinforced feedback of flows of information from the diagnosis to the therapy is the representation of the novelty of the personalized nanomedicine, which offers the opportunity to enhance the efficacy of a drug using patient-specific knowledge.

Missing clinical data in the literature related to coefficients needed for the quantification of selected stocks, flows, and processes did not permit yet to simulate the dynamic behavior of the system using a simulator, as analytical and computational models require the use of specific inventories on clinical data (Romano et al., 2021). However, the presented ST diagram allows us to investigate system configurations in response to external driving forces, with the aim at understanding and designing even safer personalized nanomedicine.

## Conclusion

This paper introduced the ST approach in nanomedicine and presented one of its applications to a real case study under preclinical stage: a dispersion of Fe_3_O_4_ NBMs coated with PEG and PLGA containing a generic anticancer drug. The proposed diagram successfully investigated the complexity of the administration of this nanotheranostic agent by adopting a personalized medicine approach.

The development of a stock-flow diagram allowed to identify interconnections between diagnosis and therapy of solid tumors after the administration of a nano-based contrast agent. The identification of these interconnections has revealed important leverage points and reinforcing feedbacks which permits to represent the complexity of the investigated system.

The application of the ST theory in nanomedicine using a case study that is still under preclinical stage offers the opportunity to focus on the novelty of the approach rather than the case study itself, focusing on the potential of a complementary approach to set up theranostic procedures.

Besides the use of stock-flow diagrams to point out the tight relationship between the diagnostic and therapeutic aspects after the administration of a theranostic agent, the presented approach addresses some peculiar development prospects. In particular, physiological responses and their feedbacks during theranostic procedures are expected to depend on the relative time scales of the processes in the systems. Indeed, the optimization of diagnostic and therapeutic actions may require trade-off in the operation planning of the administration of nano-based theranostic agents.

In this sense, the possibility of developing a quantitative simulator based on the stock-flow diagram makes the approach suitable to be personalized to specific agents and specific patients once clinical data on the administration of nanotheranostic agents will be available.

Finally, it is also worth mentioning how the presented approach can address a suitable standardization for the description and communication of theranostic activities, potentially useful in both in their development and their dissemination.

## Data Availability Statement

The raw data supporting the conclusions of this article will be made available by the authors, without undue reservation.

## Author Contributions

VC: conceptualization and writing – original draft preparation. FG: methodology, writing – review, and editing. AR: investigation and supervision. All authors contributed to the article and approved the submitted version.

## Conflict of Interest

The authors declare that the research was conducted in the absence of any commercial or financial relationships that could be construed as a potential conflict of interest.

## References

[B1] Aggarwal V. Tuli H. S. Varol A. Thakral F. Yerer M. B. Sak K. (2019). Role of reactive oxygen species in cancer progression: molecular mechanisms and recent advancements. *Biomolecules* 9:735. 10.3390/biom9110735 31766246 PMC6920770

[B2] Aires A. Cabrera D. Alonso-Pardo L. C. Cortajarena A. L. Teran F. J. (2017). Elucidation of the physicochemical properties ruling the colloidal stability of iron oxide nanoparticles under physiological conditions. *ChemNanoMat.* 3 183–189. 10.1002/cnma.201600333

[B3] Alshehri S. Imam S. S. Rizwanullah M. Akhter S. Mahdi W. Kazi M. (2021). Progress of cancer nanotechnology as diagnostics, therapeutics, and theranostics nanomedicine: preclinical promise and translational challenges. *Pharmaceutics* 13 1–35. 10.3390/pharmaceutics13010024 33374391 PMC7823416

[B4] Ansari M. O. Ahmad M. F. Shadab G. G. H. A. Siddique H. R. (2018). Superparamagnetic iron oxide nanoparticles based cancer theranostics: a double edge sword to fight against cancer. *J. Drug Delivery Sci. Technol.* 45 177–183. 10.1016/j.jddst.2018.03.017

[B5] Assaraf O. B. Z. Orion N. (2005). Development of system thinking skills in the context of earth system education. *J. Res. Sci. Teach.* 42 518–560. 10.1002/tea.20061

[B6] Balti A. Zugaj D. Fenneteau F. Tremblay P. O. Nekka F. (2021). Dynamical systems analysis as an additional tool to inform treatment outcomes: the case study of a quantitative systems pharmacology model of immuno-oncology. *Chaos* 31:023124. 10.1063/5.0022238 33653032

[B7] Bielekova B. Vodovotz Y. An G. Hallenbeck J. (2014). How implementation of systems biology into clinical trials accelerates understanding of diseases. *Front. Neurol.* 5:102. 10.3389/fneur.2014.00102 25018747 PMC4073421

[B8] Brown M. T. (2004). A picture is worth a thousand words: energy systems language and simulation. *Ecol. Model.* 178 83–100. 10.1016/j.ecolmodel.2003.12.008

[B9] Bruschi M. L. de Toledo L. (2019). Pharmaceutical applications of iron-oxide magnetic nanoparticles. *Magnetochemistry* 5:50. 10.3390/magnetochemistry5030050

[B10] Chelliah V. Lazarou G. Bhatnagar S. Gibbs J. P. Nijsen M. Ray A. (2021). Quantitative systems pharmacology approaches for immuno-oncology: adding virtual patients to the development paradigm. *Clin. Pharmacol. Ther.* 109 605–618. 10.1002/cpt.1987 32686076 PMC7983940

[B11] Comte B. Baumbach J. Benis A. Basílio J. Debeljak N. Flobak Å, et al. (2020). Network and systems medicine: position paper of the european collaboration on science and technology action on open multiscale systems medicine. *Network Syst. Med.* 3 67–90. 10.1089/nsm.2020.0004 32954378 PMC7500076

[B12] Cooper G. M. (2000). *The Cell: A Molecular Approach*, 2nd Edn. Sunderland, MA: Sinauer ssociates.

[B13] Dadfar S. M. Roemhild K. Drude N. I. von Stillfried S. Knüchel R. Kiessling F. (2019). Iron oxide nanoparticles: diagnostic, therapeutic and theranostic applications. *Adv. Drug Delivery Rev.* 138 302–325. 10.1016/j.addr.2019.01.005 30639256 PMC7115878

[B14] Degrauwe N. Hocquelet A. Digklia A. Schaefer N. Denys A. Duran R. (2019). Theranostics in interventional oncology: versatile carriers for diagnosis and targeted image-guided minimally invasive procedures. *Front. Pharmacol.* 10:450. 10.3389/fphar.2019.00450 31143114 PMC6521126

[B15] Douziech-Eyrolles L. Marchais H. Hervé K. Munnier E. Soucé M. Linassier C. (2007). Nanovectors for anticancer agents based on superparamagnetic iron oxide nanoparticles. *Int. J. Nanomed.* 2 541–550. PMC2676819 18203422

[B16] European Commission (2007). *Regulation (EC) No 1394/2007 OF THE EUROPEAN PARLIAMENT AND OF THE COUNCIL of 13 November 2007 on Advanced Therapy Medicinal Products and Amending Directive 2001/83/EC and Regulation (EC) No 726/2004.* Brussells: European Commission.

[B17] Forrester J. W. (1971). Counterintuitive behavior of social systems. *Technol. Forecasting Soc. Change* 3 1–22. 10.1016/S0040-1625(71)80001-X

[B18] Galli F. Varani M. Lauri C. Silveri G. G. Onofrio L. Signore A. (2021). Immune cell labelling and tracking: implications for adoptive cell transfer therapies. *EJNMMI Radiopharmacy Chem.* 6:7. 10.1186/s41181-020-00116-7 33537909 PMC7859135

[B19] Ghazanfari M. R. Kashefi M. Shams S. F. Jaafari M. R. (2016). Perspective of Fe3O4 nanoparticles role in biomedical applications. *Biochem. Res. Int.* 2016:7840161. 10.1155/2016/7840161 27293893 PMC4884576

[B20] Gonella F. Casazza M. Cristiano S. Romano A. (2020). Addressing COVID-19 communication and management by a systems thinking approach. *Front. Commun.* 5:63. 10.3389/fcomm.2020.00063

[B21] Goya G. F. Lima E. Arelaro A. D. Torres T. Rechenberg H. R. Rossi L. (2008). Magnetic hyperthermia with Fe3O4 nanoparticles: the influence of particle size on energy absorption. *IEEE Trans. Magnetics* 44(11 Pt 2) 4444–4447. 10.1109/TMAG.2008.2003508

[B22] Haraldsson H. V. (2004). *Introduction to System Thinking and Causal Loop Diagrams* (Issue October). Lund: Lund University. 10.1007/978-981-10-2045-2_3

[B23] Haraldsson H. V. Sverdrup H. (2013). “Finding simplicity in complexity in biogeochemical modelling,” in *Environmental Modelling: Finding Simplicity in Complexity: Second Edition, August*, eds Wainwright J. Mulligan M. (Hoboken, NJ: Wiley), 277–289. 10.1002/9781118351475.ch17

[B24] Higgins K. L. (2015). *Economic Growth and Sustainability. Systems Thinking for a Complex World.* Amsterdam: Elsevier Inc.

[B25] Hou C. H. Lin F. L. Hou S. M. Liu J. F. (2014). Hyperthermia induces apoptosis through endoplasmic reticulum and reactive oxygen species in human Osteosarcoma cells. *Int. J. Mol. Sci.* 15 17380–17395. 10.3390/ijms151017380 25268613 PMC4227168

[B26] Jagtap J. M. Parchur A. K. Sharma G. (2020). “Smart nanomaterials for tumor targeted hyperthermia,” in *Intelligent Nanomaterials for Drug Delivery Applications*, eds Ahmad N. Gopinath P., (Amsterdam: Elsevier), 10.1016/b978-0-12-817830-0.00003-5

[B27] Keek S. A. Leijenaar R. T. H. Jochems A. Woodruff H. C. (2018). A review on radiomics and the future of theragnostics for patient selection in precision medicine. *Br. J. Radiol.* 91 1–16. 10.1259/bjr.20170926 PMC6475933 29947266

[B28] Kutty A. A. Abdella G. M. Kucukvar M. Onat N. C. Bulu M. (2020). A system thinking approach for harmonizing smart and sustainable city initiatives with United Nations sustainable development goals. *Sustainable Dev.* 28 1347–1365. 10.1002/sd.2088

[B29] Lagziel S. Lee W. D. Shlomi T. (2019). Studying metabolic flux adaptations in cancer through integrated experimental-computational approaches. *BMC Biol.* 17:51. 10.1186/s12915-019-0669-x 31272436 PMC6609376

[B30] Le Q. V. Yang G. Wu Y. Jang H. W. Shokouhimehr M. Oh Y. K. (2019). Nanomaterials for modulating innate immune cells in cancer immunotherapy. *Asian J. Pharm. Sci.* 14 16–29. 10.1016/j.ajps.2018.07.003 32104435 PMC7032173

[B31] Lee J. M. Gianchandani E. P. Papin J. A. (2006). Flux balance analysis in the era of metabolomics. *Brief. Bioinform.* 7 140–150. 10.1093/bib/bbl007 16772264

[B32] Liou G. Y. Storz P. (2010). Reactive oxygen species in cancer. *Free Radical Res.* 44:10.3109/10715761003667554. 10.3109/10715761003667554 20370557 PMC3880197

[B33] Meadows D. H. (2008). “Thinking in systems: a primer,” in *Sustainability Institute*, ed. Wright D. (White River Junction, VT: Chelsea Green publishing).

[B34] Mura S. Couvreur P. (2012). Nanotheranostics for personalized medicine. *Adv. Drug Delivery Rev.* 64 1394–1416. 10.1016/j.addr.2012.06.006 22728642

[B35] Nuzhina J. V. Shtil A. A. Prilepskii A. Y. Vinogradov V. V. (2019). Preclinical evaluation and clinical translation of magnetite-based nanomedicines. *J. Drug Delivery Sci. Technol.* 54:101282. 10.1016/j.jddst.2019.101282

[B36] Odum H. Odum E. (2000). *Modeling for All Scales.* Cambridge, MA: Academic Press.

[B37] Revia R. A. Zhang M. (2016). Magnetite nanoparticles for cancer diagnosis, treatment, and treatment monitoring: recent advances. *Mater. Today* 19 157–168. 10.1016/j.mattod.2015.08.022 27524934 PMC4981486

[B38] Romano A. Casazza M. Gonella F. (2021). Addressing non-linear system dynamics of single-strand RNA virus–host interaction. *Front. Microbiol.* 11:254. 10.3389/fmicb.2020.600254 33519741 PMC7843927

[B39] Ryu J. H. Lee S. Son S. Kim S. H. Leary J. F. Choi K. (2014). Theranostic nanoparticles for future personalized medicine. *J. Controlled Release* 190 447–484. 10.1016/j.jconrel.2014.04.027 24780269

[B40] Singh D. Dilnawaz F. Sahoo S. K. (2020). Challenges of moving theranostic nanomedicine into the clinic. *Nanomedicine (Lond)* 15 111–114. 10.2217/nnm-2019-0401 31903854

[B41] Slimen I. B. Najar T. Ghram A. Dabbebi H. Ben Mrad M. Abdrabbah M. (2014). Reactive oxygen species, heat stress and oxidative-induced mitochondrial damage. A review. *Int. J. Hyperthermia* 30 513–523. 10.3109/02656736.2014.971446 25354680

[B42] Sterman J. D. (2000). *Business Dynamics: Systems Thinking and Modeling for a Complex World.* New York, NY: McGraw-Hill.

[B43] Sun D. Zhou S. Gao W. (2020). What went wrong with anticancer nanomedicine design and how to make it right. *ACS Nano* 14 12281–12290. 10.1021/acsnano.9b09713 33021091

[B44] Theek B. Rizzo L. Y. Ehling J. Kiessling F. Lammers T. (2014). The theranostic path to personalized nanomedicine. *Clin. Transl. Imaging* 2 67–76. 10.1007/s40336-014-0051-5 24860796 PMC4031631

[B45] Vallabani N. V. S. Singh S. (2018). Recent advances and future prospects of iron oxide nanoparticles in biomedicine and diagnostics. *3 Biotech* 8 1–23. 10.1007/s13205-018-1286-z 29881657 PMC5984604

[B46] Verry C. Dufort S. Lemasson B. Grand S. Pietras J. Troprès I. (2020). Targeting brain metastases with ultrasmall theranostic nanoparticles, a first-in-human trial from an MRI perspective. *Sci. Adv.* 6:eaay5279. 10.1126/sciadv.aay5279 32832613 PMC7439298

[B47] Wang H. Sové R. J. Jafarnejad M. Rahmeh S. Jaffee E. M. Stearns V. (2020). Conducting a virtual clinical trial in HER2-negative breast cancer using a quantitative systems pharmacology model with an epigenetic modulator and immune checkpoint inhibitors. *Front. Bioeng. Biotechnol.* 8:141. 10.3389/fbioe.2020.00141 32158754 PMC7051945

[B48] Wang X. M. Ramalingam M. Kong X. Zhao L. (2018). *Nanobiomaterials. Classification, Fabrication and Biomedical Applications.* Weinheim: Wiley-VCH.

[B49] World Health Organization (WHO) (2009). *Systems Thinking for Health Systems Strenghtening*. *November.* Geneva: World Health Organization.

[B50] Yu M. Zheng J. (2015). Clearance pathways and tumor targeting of imaging nanoparticles. *ACS Nano* 9 6655–6674. 10.1021/acsnano.5b01320.Yu 26149184 PMC4955575

